# Organic Solvent Sensors Using Polymer-Dispersed Liquid Crystal Films with a Pillar Pattern

**DOI:** 10.3390/polym13172906

**Published:** 2021-08-29

**Authors:** Chia-Yi Huang, Shih-Hung Lin

**Affiliations:** 1 Department of Applied Physics, Tunghai University, Taichung 40704, Taiwan; chiayihuang@thu.edu.tw; 2 Department of Optometry, Chung Shan Medical University, Taichung 40201, Taiwan; 3 Department of Ophthalmology, Chung Shan Medical University Hospital, Taichung 40201, Taiwan

**Keywords:** sensor, polymer-dispersed liquid crystal (PDLC), organic solvent

## Abstract

An organic solvent sensor of polymer-dispersed liquid crystals (PDLCs) film is fabricated by a combination of tri-functional monomers and LCs. When the patterned PDLC film comes into contact with the organic solvent, the organic solvent will penetrate into the film to induce the orientation of the liquid crystals, which will change from an ordered to a disordered state, which causes the PDLC film to scatter incident light. The experiment used acetone and ethanol as the organic solvents of interest. The results show that the patterned PDLC film has a stronger response to acetone than to ethanol. Based on the difference in the intensity of light scattering and the response time of the patterned PDLC film to different organic solvents, the results can be used to identify and recognize different types of organic solvents.

## 1. Introduction

The combination of polymers and liquid crystals (LCs) can be classified into different configurations via the properties of the polymers, including LC cells with photoconductive polymer films [1,2], LC cells with polymer structures [3,4], polymer-stabilized LCs (PSLCs) [5,6,7], polymer-dispersed LCs (PDLCs) [8,9,10,11,12,13,14,15,16,17,18], and LC elastomers [19]. PDLC films that comprise LC droplets dispersed in polymers are formed due to the light-induced phase separation in the precursors of the PDLCs. The PDLC films are opaque at zero voltage because the mismatching of the refractive indices of the LC droplets and polymers scatter the lights that are incident to the films [8,9,10,11,12,13,14]. Application of voltages to the PDLC films makes them transparent due to the matching of the refractive indices of the LC droplets and polymers [8,9,10,11,12,13,14]. The transmittances of the PDLC films can be modulated by external voltages, and polarizers are not required at the transmittance modulation. Therefore, PDLC films have potential in developing electro-optic devices such as displays [8,9,10,11,12], lenses [13,14], sensors [15,16], gratings [17,18], lasers [20,21,22], and light modulators [23,24,25].

The cross-linked polymers consisting of mono- and bi-functional monomers have smaller anchoring energies than those consisting of tri-functional monomers, so the mono- and bi-functional monomers are widely used to fabricate the PDLC films with small operating voltages and large contrast ratios. Therefore, the PDLC films that involve mono- and bi-functional monomers can be used for displays, electric curtains, and electronic labels. The cross-linked polymers consisting of tri-functional monomers have excellent mechanical properties against compression, stretching, and bending. Therefore, it is of great interest to researchers to develop PDLC films.

This work used a tri-functional monomer and LCs to fabricate a PDLC film via photopolymerization, and the film was patterned by photolithography and imprinting. As the PDLC film with a pillar pattern is exposed to acetone, acetone will penetrate into the film to induce the disordered orientation of LC molecules in acetone, resulting in the scattering of incident light. A PDLC film without a pillar pattern was used to evaluate the response of the PDLC film with the pillar pattern to acetone. The PDLC film with the pillar pattern had a lager change in its transmittance than the PDLC film without a pillar pattern after the two films were exposed to acetone. The PDLC film with the pillar pattern had a weaker response to ethanol than to acetone. Therefore, the PDLC film with the pillar pattern can be used to develop solvent sensors and has potential in sensing gas.

## 2. Materials and Methods

**Figure 1:**
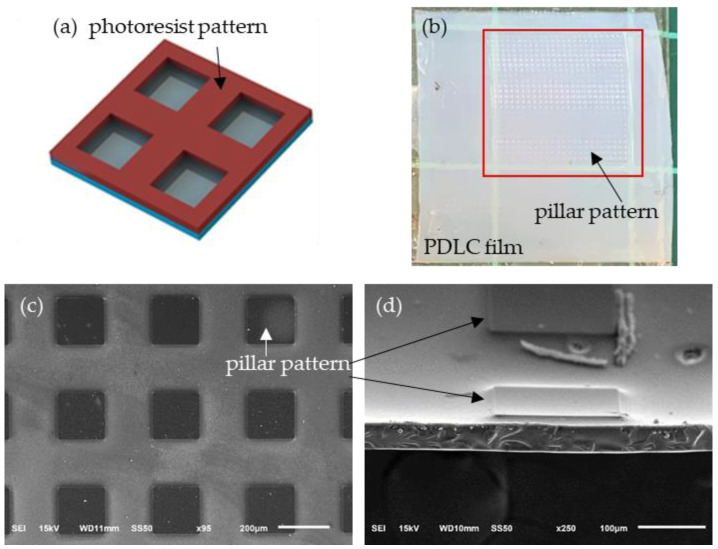
(**a**) Schematic drawing of glass substrate with photoresist pattern; (**b**) image of patterned PDLC film; (**c**) SEM image of top view of patterned PDLC film; (**d**) SEM image of section view of patterned PDLC film.

Figure 1a presents the schematic drawing of a glass substrate with a photoresist grid pattern. This work used photolithography to make a grid pattern on glass substrates. A photoresist (EPG516, Everlight Chemical Industrial Co., Taiwan) was spin-coated on a glass substrate with an area of 2 cm × 2 cm. The glass substrate with the photoresist layer was put on a hot plate for soft baking. A grid pattern on a photomask was transferred onto the photoresist layer under UV irradiation. The irradiated photoresist layer was developed using a developer (EPD48, Everlight Chemical Industrial Co., Tainan, Taiwan) and then was fixed using water. The photoresist grid pattern was obtained following the fixing process. The photoresist grid pattern was deposited on the glass substrate and had a line width and thickness of 200 μm and 11 μm, respectively. Polyimide was coated on the glass substrates with the photoresist patterns and rubbed to generate anchoring in the polyimide surface. Each of the empty cells was fabricated by the glass substrate with polyimide-coated photoresist patterns and another glass substrate with a rubbed polyimide layer. The two glass substrates were separated by two plastic spacers with a thickness of 25 μm or 50 μm. A PDLC precursor consisted of a nematic LC HTW114200-100 (65 wt.%, Fusol Material Co., Tainan, Taiwan), monomer TMPTA (33 wt.%, Sigma-Aldrih, St. Louis, MI, USA), and photoinitiator IRG 184 (2 wt.%, Ciba, Basel, Switzerland). The LC has extraordinary and ordinary refractive indices of 1.77 and 1.51, respectively, at 25 °C. The TMPTA has a refractive index of 1.47 at 25 °C. The empty cells were filled with the PDLC precursor, and then the PDLC precursor was cured using UV light with a center wavelength of 365 nm and an intensity of 5 mW/cm^2^ for 50 min. The temperature of the cells was kept at 13 °C during the curing. After the two glass substrates were separated and removed from the cell, the patterned PDLC film could be directly taken out and used. Figure 1b presents the image of the patterned PDLC film with an area of 1.5 cm × 1.5 cm. Figure 1c,d shows the SEM image of the patterned PDLC film. In Figure 1c, the 200 μm × 200 μm square is the PDLC pillar pattern. The cross-section of the patterned PDLC film presents the thickness of the film, which was about 40–50 μm, and the height of the pillar pattern was about 10 μm, as shown in Figure 1d.

**Figure 2:**
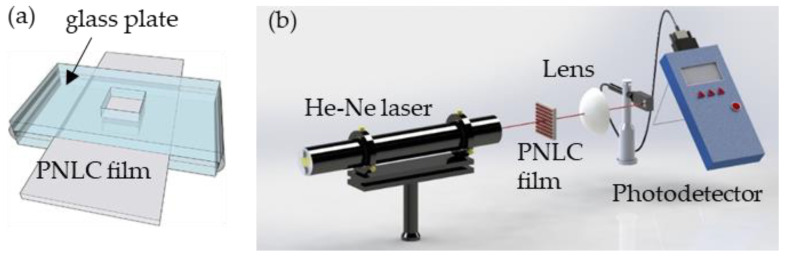
(**a**) PDLC film framed by glass plate with a square hole with a side length of 1.0 cm; (**b**) measurement of transmittances of patterned and planar PDLC films that are exposed to organic solvents.

Each of the patterned and planar PDLC films was framed using a glass plate with a square hole with a side length of 1.0 cm, as presented in Figure 2a. Organic solvents with a volume of 10 μL were dropped on these films. A probe light from a He-Ne laser with a wavelength of 632.8 nm was used to study the effect of organic solvents on the PDLC films, as presented in Figure 2b. The probe light was normally incident to each of these films, and a lens was placed behind that film to collect the light that passes through them. A power meter (NOAII, Ophir, Jerusalem, Israel) was placed behind the lens to detect the intensity of the transmitting light.

## 3. Results and Discussion

**Figure 3:**
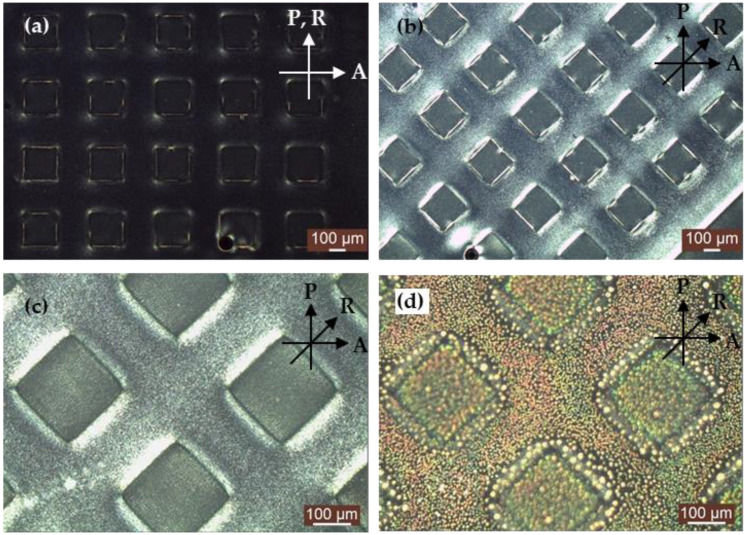
Microscopic images of patterned PDLC films under crossed polarizers at (**a**) R//P; (**b**) 45° between R and P; (**c**) without acetone; (**d**) with acetone. P and A denote the transmission axes of the polarizer and analyzer, respectively, and R denotes the rubbing direction.

Figure 3a,d depict polarizing microscopic images of patterned PDLC films with planar alignment. Figure 3a shows that when the rubbing direction and the polarizer are at an angle of 0 degree, because the LC orientation is parallel to the direction of the polarizer, the incident light cannot pass through the analyzer, and the image of patterned PDLC film exhibit a dark state. When the rubbing direction and the polarizer have an angle of 45 degrees, the LC orientation was not parallel to the direction of the polarizer, causing part of the light to pass through the analyzer, and the image of patterned PDLC film appears in a bright state, as shown in Figure 3b. Figure 3c shows the PDLC film without exposure to acetone. The alignment direction of patterned PDLC film was at a 45-degree angle with the polarizer, and the LC molecules were arranged in an orderly nematic phase. When the PDLC film was exposed to acetone, the binding force between the molecules in the acetone was reduced, and the overall ordered molecules were split into the LC droplets of the disordered phase. The disordered LC droplets caused strong light scattering, as shown in Figure 3d.

**Figure 4:**
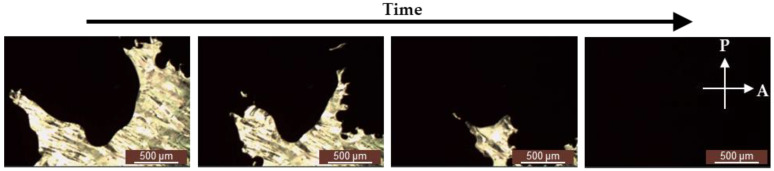
Dynamic images of nematic LCs that were exposed to acetone by a polarizing optical microscope under crossed polarizers.

A nematic LC cell was placed in a polarizing optical microscope under crossed polarizers, and a camera was used to capture the dynamic images of the nematic LC that was exposed to acetone. Figure 4 presents the dynamic images of the nematic LC that was exposed to acetone by a polarizing optical microscope under crossed polarizers. The leftmost image of Figure 4 presents that the dark area refers to acetone, and the bright area stands for the LC crystalline. Throughout the time course, when acetone was distributed over the nematic LCs, acetone existed between the LC molecules. Therefore, the reduced intermolecular force of the LC molecules loosened the LC crystalline. That explains why the bright area turns to dark—because the LCs from a liquid crystalline phase changed from ordered to disordered. Figure 4 verifies that the interaction mechanism of the nematic LCs and acetone was the phase transition of the LCs from a liquid crystalline phase to a disordered phase in the presence of acetone.

**Figure 5:**
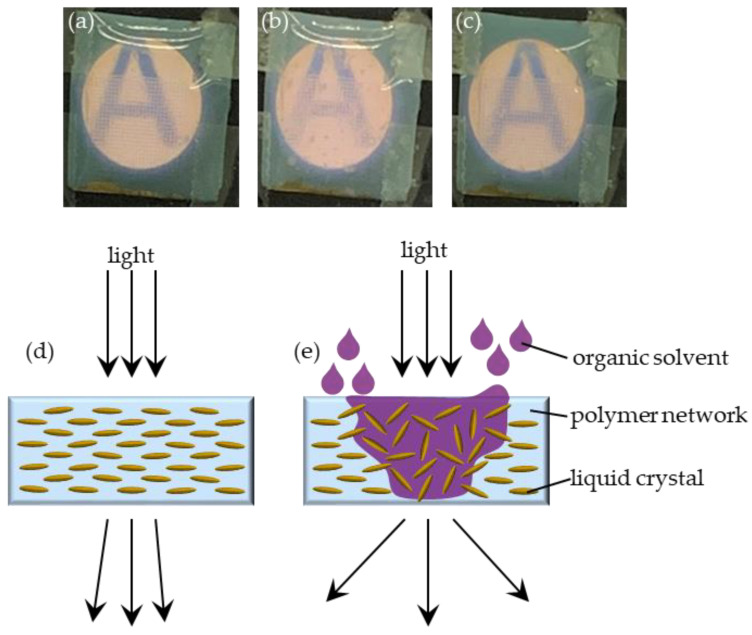
Images of patterned PDLC film (**a**) before exposure to acetone; (**b**) during exposure to acetone; (**c**) after complete volatilization of acetone; (**d**,**e**) mechanism of patterned PDLC film in organic solvent.

Figure 5a–c demonstrates the images of a patterned PDLC film before exposure to acetone, in exposure to acetone, and after the complete volatilization of acetone. The four corners of the patterned PDLC film were fixed on a metal plate with a circular hole with a diameter of 1 cm using 3M tapes. The letter “A” was printed on a paper, and the paper was placed behind the metal plate. Figure 5a presents that the letter-A image was clear before the patterned PDLC film was exposed to acetone. Figure 5b displays that the letter-A image was unclear when the patterned PDLC film was exposed to acetone. The unclear image arose because acetone disturbs the orientation of the LCs of the patterned PDLC film. Figure 5d,e demonstrates the mechanism of the patterned PDLC film in acetone. When the PDLC film was not exposed to organic solvent, the LC molecules exhibited a more ordered orientation, as shown in Figure 5d. Figure 5e presents that the PDLC film was exposed to organic solvent where the organic solvent penetrates into the PDLC film. The organic solvent molecules penetrated and accumulated in the cross-linked polymer, followed by disturbing the alignment of the LC molecules, which resulted in disordering the orientations of the LC molecules. The disordered orientations of LC molecules induced the light scattering. In other words, the intermolecular force of the LCs was decreased in the acetone. The decreased intermolecular force disordered the orientations of the LC molecules, scattering the light that blurs the A of Figure 5b. When the volatilization of acetone was more significant than the penetration of acetone in the PDLC film, the LCs returned to the initial orientations due to the loss of the interference of acetone molecules and the original alignment of the cross-linked polymer. Therefore, the letter-A image in Figure 5c was clear after the complete volatilization of acetone.

**Figure 6:**
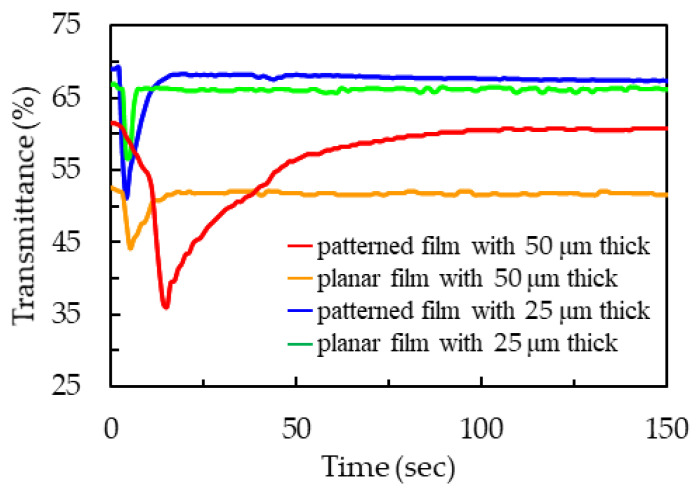
Time-dependent transmittance curves of the PDLC films with different thicknesses and surfaces that were exposed to acetone.

Figure 6 presents the time-dependent transmittance curve *T*(*t*) of the PDLC films with different thicknesses and surfaces that were exposed to acetone. The transmittances of the films were defined as a ratio of the intensity of the light that passes through the film to the light that is incident to the film. When the PDLC film was exposed to acetone, the penetration of acetone into film was greater than the volatilization of acetone. Acetone disturbs the original orientation of LC molecules, causing the LC orientation to become disordered.

The red line in Figure 6 presents the time-dependent transmittance curve *T*(*t*) of the patterned PDLC film with a thickness of 50 μm that is exposed to acetone. When the penetration of acetone into film was greater than the volatilization of acetone, the transmittance of the patterned PDLC film decreased from its initial value of 61.5% to a minimum value of 36.1% in a short duration from t = 0 s to t = 15 s. The scattering of the PDLC film resulted in a difference in transmittance of 25.4%. Subsequently, the volatilization of acetone was more significant than the penetration of acetone, and the transmittance increased from the minimum value to the initial value in a long duration from t = 15 s to t = 96 s. The time for the decrease/increase in the transmittance from the initial/minimum value to the minimum/initial value was 15/81 s, which was named the penetration/volatilization time of acetone. The orange line in Figure 6 presents the time-dependent transmittance curve *T*(*t*) of the planar PDLC film with a thickness of 50 μm that was exposed to acetone. Acetone had a longer penetration time in the patterned PDLC film than in the planar PDLC film. In addition, acetone had a longer penetration time in the patterned PDLC film than in the planar PDLC film. Therefore, the patterned PDLC film exhibited a stronger response to acetone than the planar PDLC film. The strong response to acetone was caused by the large surface area of the patterned PDLC film. The acetone molecules had aa larger contact area on the surface and penetrate into the patterned PDLC film rather than the surface of the planar PDLC film because the former exhibits a stronger response to acetone than the latter. Therefore, acetone had a longer volatilization time in the patterned PDLC film than in the planar PDLC film.

The blue line in Figure 6 presents the time-dependent transmittance curve *T*(*t*) of the patterned PDLC film with a thickness of 25 μm that was exposed to acetone. When the penetration of acetone into film was greater than the volatilization of acetone, the transmittance of the patterned PDLC film decreased from its initial value of 68.9% to a minimum value of 51.1% in a short duration from t = 0 s to t = 4 s. The scattering of the PDLC film resulted in a difference in transmittance of 17.8%. Subsequently, the volatilization of acetone was more significant than the penetration of acetone, and the transmittance increased from the minimum value to the initial value in a long duration from t = 4 s to t = 19 s. The time for the decrease/increase in the transmittance from the initial/minimum value to the minimum/initial value was 4/15 s, which was named the penetration/volatilization time of acetone. The green line in Figure 6 presents the time-dependent transmittance curve *T*(*t*) of the planar PDLC film with a thickness of 25 μm that was exposed to acetone. From the comparison between the blue line and the green line in Figure 6, the patterned PDLC film had a larger change in the transmittance than the planar PDLC film in the penetration time. Therefore, the patterned PDLC film exhibited a stronger response to acetone than the planar PDLC film.

From the comparison between the red line and the blue line in Figure 6, it can be clearly seen that the PDLC film with 25 μm thickness was thinner, resulting in a weak light scattering response to acetone and a short response time, which makes it difficult to identify organic solvents. The PDLC film with 50 μm thick was thicker, and the light scattering response of the film was more significant than that of the blue line in Figure 6. Therefore, the following measurement experiments all used a 50-μm thick spacer to prepare the PDLC film.

**Figure 7:**
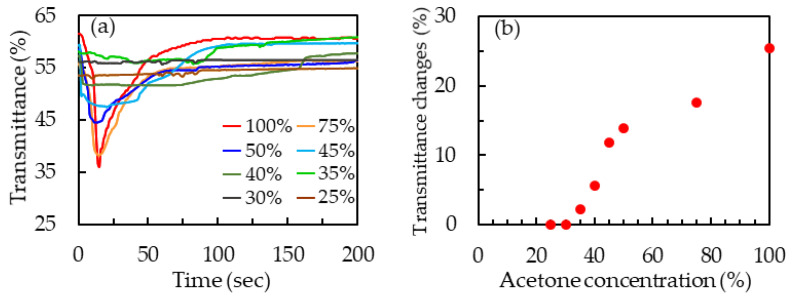
(**a**) Time-dependent transmittance curves of 50-μm-thick patterned PDLC films that were exposed to acetone solutions with concentrations of 25 v%, 30 v%, 35 v%, 40 v%, 45 v%, 50 v%, 75 v%, and 100 v%. (**b**) Transmittance changes of 50-μm-thick patterned PDLC films at acetone solutions with various concentrations.

Figure 7a presents the time-dependent transmittance curves of 50-μm-thick patterned PDLC films that were exposed to acetone solutions with concentrations of 25 v%, 30 v%, 35 v%, 40 v%, 45 v%, 50 v%, 75 v%, and 100 v%. The patterned PDLC film had a constant transmittance at the 30 v% acetone solution. Therefore, the limit of detection (LOD) of 50-μm-thick patterned PDLC film was 35 v% in this work. The transmittance change of a patterned PDLC film was used to evaluate the response of the film to an organic solvent and was defined as (*T*_i_ − *T*_m_), where *T*_i_ and *T*_m_ were the initial and the minimum transmittances of the film. Figure 7b displays the transmittance changes of the 50-μm-thick patterned PDLC films at the acetone solutions with the various concentrations. The transmittance changes of the patterned PDLC films were larger than zero as the concentrations of the acetone solutions exceeded 30 v%. Therefore, the 50-μm-thick patterned PDLC films had an LOD of 35 v% in this work.

**Figure 8:**
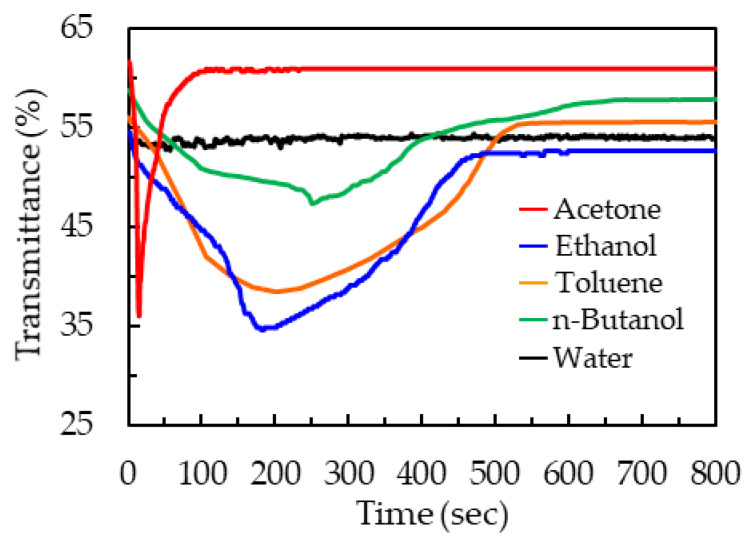
Time-dependent transmittance curves of the patterned PDLC films that were exposed to acetone, ethanol, toluene, n-butanol, and water.

Besides acetone, 50-μm-thick patterned PDLC films were used to detect ethanol, toluene, n-butanol, and water. Figure 8 presents the time-dependent transmittance curves *T*(*t*) of the 50-μm-thick patterned PDLC films that were exposed to acetone, ethanol, toluene, n-butanol, and water. When the patterned PDLC film had just been exposed to organic solvents, the penetration of organic solvents prevailed over the volatilization of organic solvents. The disturbance of organic solvents caused the orientation of LC molecules to be disordered. The transmittance decreased due to scattering of the PDLCs. Subsequently, the volatilization of organic solvents was more significant than the penetration of organic solvents, and therefore the transmittance increased. However, the patterned PDLC film did not generate any response to water. Until the end of the experimental measurement, the water droplets remained on the surface of the PDLC film.

Table 1 displays the comparison of the 50-μm-thick patterned PDLC films that were exposed to acetone, ethanol, toluene, n-butanol, and water. The experimental data in Table 1 were obtained from Figure 8. Table 1 reveals the response of the patterned films to the organic solvents according to their transmittance changes and penetration times of Figure 8: acetone >> ethanol >> toluene >> n-butanol. The experimental data in Table 1 reveal that the patterned PDLC film can be used to detect organic solvents and that it has potential in sensing organic gases.

## 4. Conclusions

This study successfully produced a PDLC film that can be used to detect organic solvents. The results show that the pillar structure of the PDLC film surface can increase the surface area and promote detection efficiency to organic solvents. From the difference of the light scattering intensity and response time of the patterned PDLC film to various organic solvents, it can be used as an index to identify different organic solvents. The patterned PDLC film has the potential to be used in detecting various organic solvents or organic gases in the future.

## Figures and Tables

**Table 1:** Comparison of the patterned PDLC films with a thickness of 50 μm that were exposed to acetone, ethanol, toluene, n-butanol, and water.

	Penetration Times (s)	Volatilization Times (s)	Transmittance Changes (%)
Acetone	15	81	25.4
Ethanol	185	296	19.8
Toluene	202	339	17.5
n-Butanol	250	418	11.4
Water	-	-	-

## Data Availability

The data presented in this study are available on request from the corresponding author.
